# Zimbabwe's emergency response to COVID-19: Enhancing access and accelerating COVID-19 testing as the first line of defense against the COVID-19 pandemic

**DOI:** 10.3389/fpubh.2022.871567

**Published:** 2022-07-19

**Authors:** Muchaneta Gudza-Mugabe, Kenny Sithole, Lucia Sisya, Sibongile Zimuto, Lincoln S. Charimari, Anderson Chimusoro, Raiva Simbi, Alex Gasasira

**Affiliations:** ^1^World Health Organization, Harare, Zimbabwe; ^2^Clinton Health Access Initiative, Harare, Zimbabwe; ^3^National Microbiology Reference Laboratory, Harare, Zimbabwe; ^4^Zimbabwe National Quality Assurance Program, Harare, Zimbabwe; ^5^Ministry of Health and Child Care, Harare, Zimbabwe

**Keywords:** decentralization, Ag-RDT, COVID-19, Zimbabwe, training

## Abstract

The Severe Acute Respiratory Syndrome Coronavirus 2 (SARS-CoV-2) spreads rapidly, causing outbreaks that grow exponentially within a short period before interventions are sought and effectively implemented. Testing is part of the first line of defense against Corona Virus Disease of 2019 (COVID-19), playing a critical role in the early identification and isolation of cases to slow transmission, provision of targeted care to those affected, and protection of health system operations. Laboratory tests for COVID-19 based on nucleic acid amplification techniques were rapidly developed in the early days of the pandemic, but such tests typically require sophisticated laboratory infrastructure and skilled staff. In March 2020, Zimbabwe confirmed its first case of COVID-19; this was followed by an increase in infection rates as the pandemic spread across the country, thus increasing the demand for testing. One national laboratory was set to test all the country's COVID-19 suspect cases, building pressure on human and financial resources. Staff burnout and longer turnaround times of more than 48 h were experienced, and results were released late for clinical relevance. Leveraging on existing PCR testing platforms, including GeneXpert machines, eased the pressure for a short period before facing the stockout of SARs-CoV-2 cartridges for a long time, leading to work overload at a few testing sites contributing to long turnaround times. On September 11, WHO released the interim guidance to use antigen rapid diagnostic test as a diagnostic tool. The Zimbabwe laboratory pillar quickly adopted it and made plans for its implementation. The National Microbiology Reference Laboratory verified the two emergency-listed kits, the Panbio Abbott and the Standard Q, Biosensor, and they met the WHO minimum performance of ≥97% specificity and ≥80% sensitivity. Decentralizing diagnostic testing leveraging existing human resources became a game-changer in improving COVID-19 containment measures. Task shifting through training on Antigen rapid diagnostic tests (Ag-RDT) commenced, and testing was decentralized to all the ten provinces, from 1 central testing laboratory to more than 1,000 testing centers. WhatsApp platforms made it easier for data to be reported from remote areas. Result turnaround times were improved to the same day, and accessibility to testing was enhanced.

## Introduction

Laboratory testing for accurate diagnosis is the pillar of COVID-19 response worldwide (1). The laboratory is a key technical area of International Health Security, enabling countries and entities to detect infectious agents of human or animal origin, enhancing preparedness and response to infectious diseases of epidemic potential (2). It plays a critical role in detecting SARS-CoV-2 infection, case management, disease surveillance and control, and providing accurate health data for national planning and decision making (3). The gold standard for testing SARS-CoV-2 infection is the reverse transcriptase-polymerase chain reaction platform (RT–PCR). With its high sensitivity and specificity benefits, this RT-PCR test has been widely used in well-resourced settings. The dramatic increase of COVID-19 cases caused by the fast-spreading virus challenged the normal standard of laboratory testing operations worldwide, and the global demand for testing has put a substantial strain on governments and institutions (4, 5). This strained the laboratory system resources, characterized by staff burnout and longer testing turnaround times, from 48 h to more than a month for remote areas (6). This was an urgent call to scale up SARS-CoV-2 testing and make it accessible to the larger marginalized against geographical barriers. The scale-up could be achieved by employing Point-of-care testing for early disease detection with affordable testing platforms demanding less operational resources (7).

In September 2020, the World Health Organization (WHO) recommended using rapid antigen diagnostic tests in response to the challenges countries were facing (8, 9). In October 2020, Latin American countries under the Pan American Health Organization (PAHO) embarked on using Ag-RDT in marginalized areas to increase accessibility and testing capacity (10). In December 2020, the European Union adopted Ag-RDT in places with limited nucleic acid amplification test (NAAT) capacities, particularly RT-PCR assays (11). However, timely access and geographical availability to COVID-19 diagnostic testing remain a challenge in the WHO African region and other socially disadvantaged regions in developed countries (3, 12).

Zimbabwe was one of the WHO-AFRO countries struggling to meet the demand for testing due to limited resources within centralized testing at the National Microbiology Reference Laboratory (NMRL). The detection of the first COVID-19 case in Zimbabwe on the 20th of March 2020 brought fear to the population, leading to increased demand for testing. The number of people who voluntarily demanded to test because they had been in contact with an infected person increased significantly, and in addition, suspect cases were reported from health facilities and postmortem screenings before burial. This demand affected the results turnaround time, exacerbated by poor logistical support for centralized testing. Samples were incorrectly stored for days without transport to ship them to the testing laboratories. It became necessary to decentralize testing, increase accessibility and testing capacity, and reduce turnaround time. Zimbabwe, therefore, adopted the use of Ag-RDT for diagnosis, which was to be implemented at all levels of the health delivery system.

## Context

Zimbabwe has a population of 14.86 million. Administratively it comprises of ten provinces with 1,848 health facilities (Ministry of Health and Child Care, National Health Strategy 2021–2025), structured into four levels. Quaternary level with six government (Gvt) central hospitals, tertiary with eight Gvt provincial hospitals, and the secondary level has 138 Gvt, Mission, and private hospitals. Lastly, the Primary level has 1,696 Gvt rural hospitals, municipal, private, mission, urban council clinics, and Rural Health Centers (RHC). The quaternary, Tertiary, and Secondary levels have functional laboratory facilities and trained laboratory technologists. However, the primary health facilities level does not have laboratory facilities, but some have simple clinic testing areas for HIV, malaria rapid diagnostic tests, and TB microscopy. Some facilities possessed molecular testing platforms within the quaternary, tertiary, and secondary levels. GeneXpert was used for other TB, Viral Load, and Early Infant Diagnosis programs. In the private sector, some institutions were using RT-PCR for their programs. All of these were capacitated through training on using COVID-19 specific protocols, and software specific for COVID-19 testing was installed on machines.

Consistently, the primary level health facilities are the majority (92%) of the health facilities. It is important to note that the top 3 levels have skilled professionals in testing, while the primary levels do not have adequate skills for testing. Most quaternary and tertiary facilities are situated within urban and peri-urban areas, whilst most secondary and primary level facilities are situated in rural settings where most of the population (68%) of Zimbabwe reside (13). They are also situated in hard-to-reach remote areas, with communication, transport, and accessibility challenges. Capacitation of Healthcare workers in these areas would significantly increase testing capacity in the country.

## Methods

### Program plan

**Figure 1:**
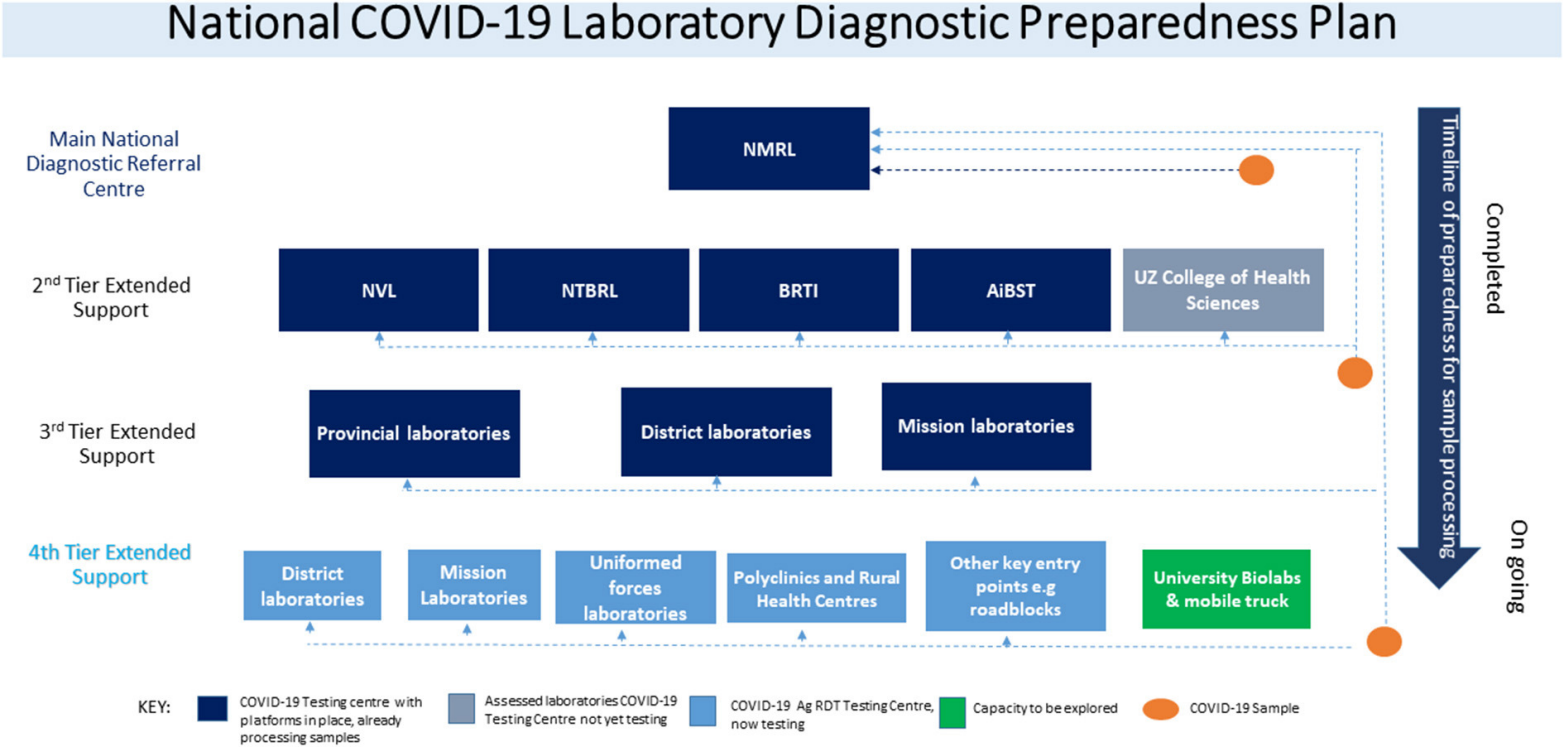
National COVID-19 Laboratory preparedness strategy plan: Tier system.

At the beginning of the COVID-19 pandemic, the MoHCC formed, nine national health strategic response pillars focused on emergency response. The following pillars were established; Coordination and monitoring, Case management, Surveillance, Ports of entry, Infection prevention and control, Risk communication and community engagement, Logistics, and procurement, and research, including the laboratory pillar. However, as the pandemic progressed, the vaccination pillar was later added to make them ten. The laboratory pillar developed the National COVID-19 testing strategy, which stratified interventions to scale up COVID-19 testing using PCR and progressively established a 4-tier system (Figure 1). The first tier represented centralized testing done only at National Microbiology Reference Laboratory. The MoHCC, through the laboratory pillar, invited the private sector with existing molecular testing capacity and technical expertise in PCR testing to expand testing. This led to the introduction of the second tier adding four private laboratories in the collaborative efforts to fight the pandemic. The third tier included a few selected provincial, district, and mission hospitals that already had GeneXpert PCR testing platforms. It must be noted that the demand for testing was classified into two categories, institutional demand, which comprised of tests demanded by health facilities and organizations, whilst community demand comprised of members of the community who wanted to be tested.

As soon as the WHO published the guideline on Ag-RDT use as a diagnostic test, the Government of Zimbabwe's MoHCC quickly adopted its use as a diagnostic tool alongside the RT-PCR platforms. The laboratory pillar reviewed the National COVID-19 Laboratory Testing Strategy and prepared the SAR-CoV-2 Ag-RDT testing implementation guideline. NMRL conducted an in-country verification exercise for the WHO emergency use listed nasopharyngeal Ag-RDT test kits Panbio (Abbott) and Standard Q (Biosensor). Satisfactory results which met the WHO guideline criteria of minimum performance requirements of ≥80% sensitivity and ≥97% specificity compared to a NAAT as a reference assay were obtained (8). The advantage of this point of care testing was that results were released within 30 min of testing, providing a quick turnaround time for patient management and contact tracing (9, 14). The first three tiers met mostly the institutional demands; community demands were greatly affected by the accessibility of testing. A fourth tier was then introduced soon after adopting the Ag-RDT testing guideline to decentralize testing to remaining provincial, district, mission, council clinics, mines, uniformed forces, universities, and rural health centers that were not testing. Consequently, MoHCC adopted task shifting as recommended by WHO guidelines to allow more efficient use of available human resources. Therefore, training was done to capacitate nurses, microscopists, and laboratory personnel at all testing centers (7, 15).

### Program implementation

In collaboration with MoHCC through the laboratory pillar, WHO and Clinton Health Access Initiative (CHAI) spearheaded the implementation of the Ag-RDT Roll-out activities. The African Society for Laboratory Medicine (ASLM), Biomedical Research and Training Institute (BRTI), and Zimbabwe National Quality Assurance Program (ZINQAP) also supported the rollout activities for this program. Early in November 2020, MoHCC, CHAI, WHO & ASLM urgently set up the first virtual zoom meeting to orient the Master trainers with the necessary information and knowledge to roll out training. Immediately after the orientation, the Master trainers scheduled and facilitated a 2-day trainer of trainers (ToTs) in mid-November 2020, 35 participants drawn from the ten provinces of Zimbabwe, selected with the guidance of MoHCC, were trained. The trained trainers were then key in the subsequent decentralization trainings to provinces and districts. Each provincial team consisted of 3 or more trainers, who further cascaded down the training in their respective areas and facilities. Each province and district hospital had a targeted 8–15 HCWs trained, that is, nurses who had been trained on HIV rapid testing and lab personnel who were pioneers in testing using COVID-19 Ag testing. The training was competence-based, consisting of theory and practical sessions on donning and doffing, sample collection, testing, interpretation, and reporting results. The training material was a comprehensive antigen-RDT training package available on the ASLM website (14). SD Biosensor and Abbott suppliers did virtual and in-person sessions during the ToTs. At the end of the training, trainees wrote a competency test part of the ASLM package for certification and a practical exam where trainees were observed performing the whole testing process.

The third stage had ten teams formed consisting of 3 trainers trained during the ToTs and one national trainer to train in each province. The training was initially conducted at the provincial hospitals and three other high-volume sites at the secondary level. An initial 40 facilities were earmarked for the initial phase, and 38 facilities had been trained by December 2020. The training was reduced to a day's training since Health Care workers (HCW) and Laboratory personnel had prior knowledge of collecting samples and had been trained on Infection Prevention and Control (IPC) modules before Antigen trainings. CHAI supplies of Panbio Abbott kits that were already in-country helped in the trainings and initial testing.

During the training, the participants were given a trainer's manual with all the 11 modules, and each testing site was supported with a Panbio Abbott kit with 25 tests for immediate testing. The same training model was used, a combination of theory and demonstration, and the same competency exams for certification were written. Additional documents supplied were the National COVID-19 register, COVID-19 testing algorithm, 1 page summary of the national laboratory COVID-19 testing strategy, and personal protective equipment.

The trained HCWs in each province formed small training teams to cascade training to districts, and the district teams, with the help of other provincial trainers and partners, cascaded training in the province and rural health centers. The trainings were progressive, with constant monitoring and follow-ups, which were managed through weekly reporting in the Provincial health executive (PHE) meetings.

Data management for the first 5 laboratories in the 1st and 2nd tier were managed through the use of the Laboratory Information Management System (LIMS) centralized at National reference laboratories for easy management of data and collection of daily statistics. As the pandemic progressed some provinces furnished with PCR machines were added to the LIMS. However, data obtained from all trained sites during and after decentralization were relayed through the use of WhatsApp online messaging platform, where each testing site would send results to the national level for consolidation at the end of each day. This reporting system became cumbersome as the number of sites increased. Data collection was then decentralized, and provinces were then tasked to receive information by WhatsApp from their respective districts and clinics. They would then populate an excel database with all names of testing sites per district in the province, then email the data to the National level to populate the national database. This lessened the burden of work for national data capturers. All these levels had their daily cut-off times for data receipt to enable information to be analyzed for the daily situational report (Sitrep). Sites that could not report would report on the next day's statistics. Go Data's real-time data management platform was piloted and scaled up ongoing.

The received data WERE analyzed using the MICROSOFT Excel SOFTWARE (VERSION 2108) to calculate testing rates per 10,000 PEOPLEusing the ZIMBABWE statistics record of 14,143,449 (tests done per day/total population × 10,000). THE average of tests done per day WAS calculated by THE end of the week, WHEN THE total number of tests PERFORMED was divided by 7 (days of the week). The testing turnaround was calculated from the day and time the sample was received in the testing lab to the time the results were released using the LIMS system.

## Results

**Figure 2:**
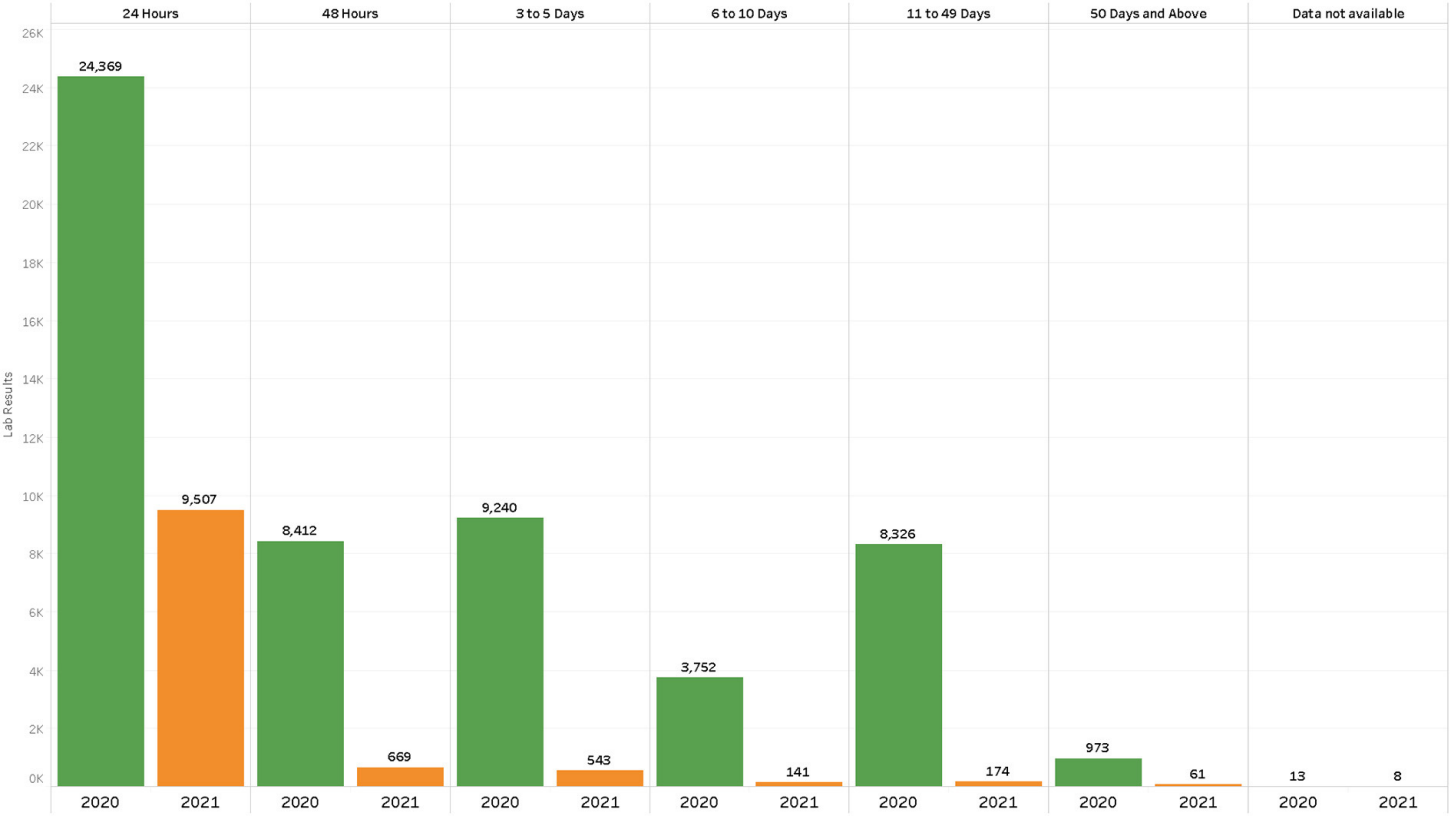
Results turnaround times July–September 2020 vs. July to September 2021 in the first five laboratories in the second tier after the decentralized testing to rural health centers.

At the beginning of the pandemic in 2020, PCR testing was conducted to confirm SARS-CoV-2 infection. In the first tier in March 2020, one central laboratory was testing, conducting an average of 350 tests per day (Table 1). The testing rates were three people per 10 000 against a target of 10 people per 10,000. PCR laboratories were added in each tier, subsequently increasing the average tests per day from 300 to 1,300 tests per day, surpassing the initial national target of 1,000 daily average tests. Testing rates increased from 5 to 8 people per 10,000 (Table 1). Testing turnaround times were incrementally showing a delay of ~50% of results to reaching the patients (Figure 2). From tier 3 to tier 4, testing sites increased from 16 to 1,500 over 10 months, while testing rates increased from 20 to 57 people per 10,000. Turnaround time for 80% of results was reduced to 0–48 h (Figure 2), meeting the desired quality standards. The testing platforms were both PCR and antigen rapid diagnostic tests (Table 1). HCWs trained to carry out COVID-19 testing increased to over 1,650.

**Table 1:** Decentralization output through the four tiers.

**Program Tier**	**Cumulative # of testing facilities**	**Cumulative # of HCW testing**	**Average # of daily tests**	**Period**	**Testing rate per 100,000 population**	**Testing platform**	**Turnaround time for 80% of Results**
1	1	4	300	March to April	3	PCR	24–72 h
2	6	12	700–800	May to June	5	PCR	24–20 days
3	16	80	800–1,300	July to Nov 2020	8	PCR	24 > 40 days
4	>1,650	>6,000	11,000	Dec to Sept 2021	20–57	PCR Ag-RDT	0–48 h

**Figure 3:**
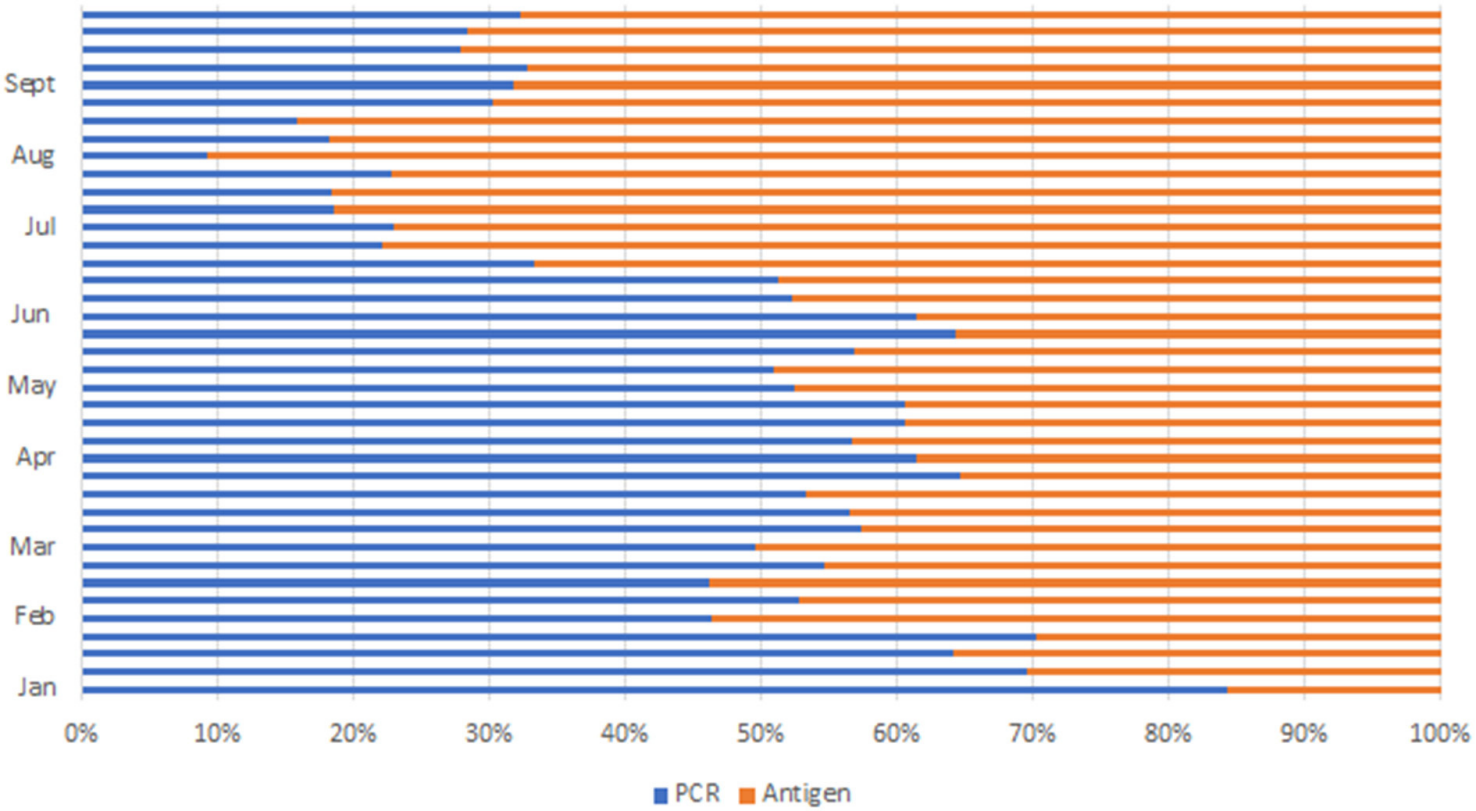
Proportion of antigen RDT tests to PCR by month in 2021.

During the peak of the first wave in August 2020, 39,682 with a daily average of 1,300 tests were conducted. After the initial rollout of antigen testing, there was a notable doubling of test statistics in December 2020 to 47,446 and further doubling to 118,361 in January 2021. The average daily test increased from 1,000 to about 4,000 tests per day. Ag-RDT platform contributed 32,685 (27%) (Figure 3) of the test performed. Test statistics increased in June and July 2021 during the peak of the third wave, with the highest number of tests conducted in week 28, where 80,250 tests were conducted, leading to an average daily test of 11,464 and monthly statistics of 285,141 tests conducted. Ag-RDT average weekly tests contributed 65,375 (81%) (Figure 3) of the tests conducted. During this period, 107 PCR laboratories were testing whilst an average of 400/470 Ag-RDT trained centers were testing and reporting.

**Figure 4:**
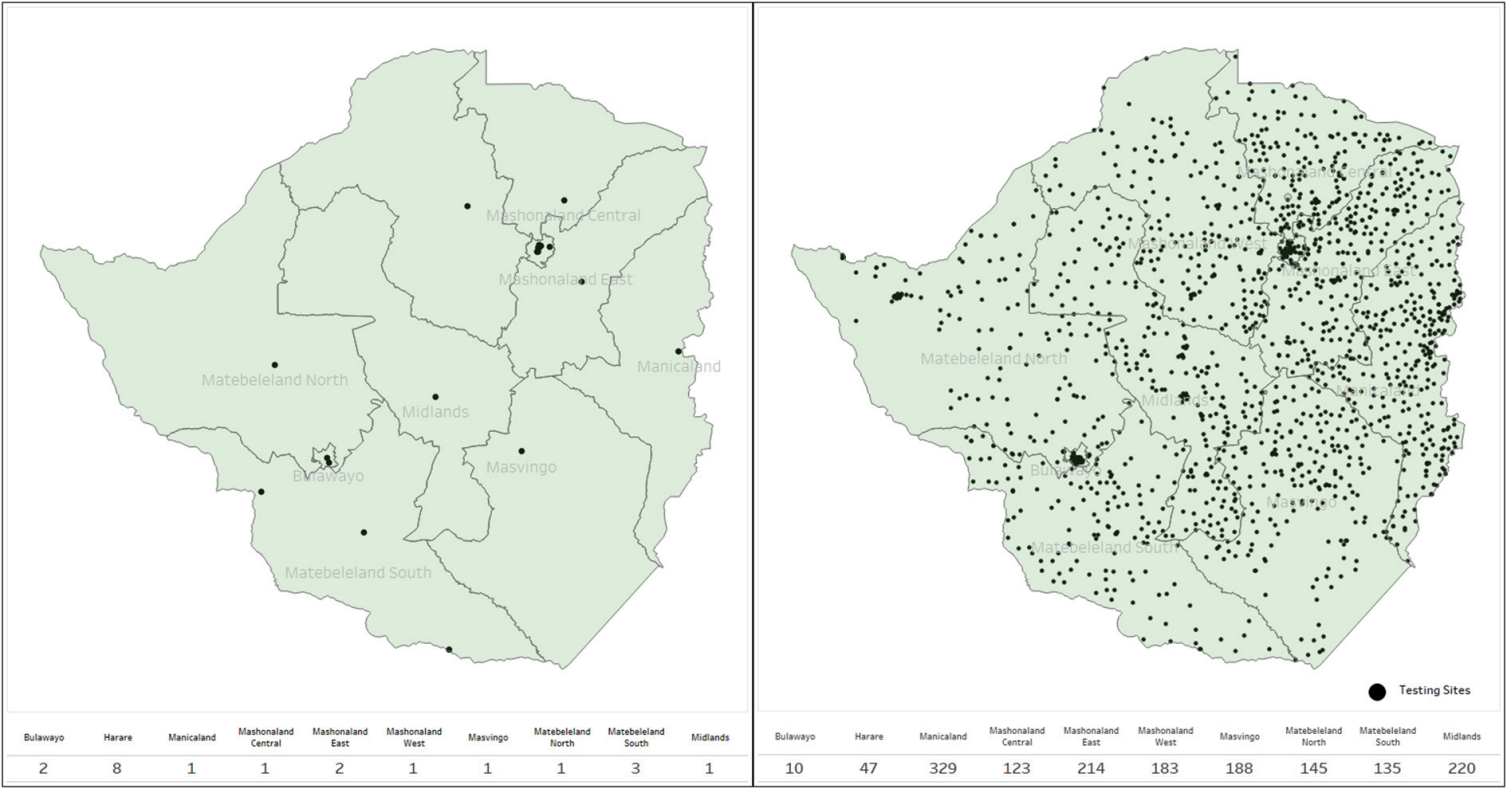
Spot map showing scale-up of testing sites increasing accessibility.

Sites were capacitated, and testing increased from the initial 38 sites to over 1,650 over 10 months, whilst health care workers trained in Ag-RDT testing increased from the initial of about 450 to over 6,000 (Figure 4). More than 200 private institutions registered and participated in COVID-19 testing and reported to the national health surveillance department, including the private laboratories, universities, mines, and uniformed forces (prisons, army, and police).

## Discussion

In Zimbabwe, testing played a key role in the national SARS-CoV-2 response through a collaboration of the public sector and private laboratories and the use of point of care (POCT) facilities. This collaboration was important in the fight against SARS-CoV-2 infections in response to the pandemic, since government infrastructure alone would not suffice (16, 17). A combination of PCR and antigen Rapid Diagnostic tests was used. Antibody RDT were initially used for screening. However, they were non-specific due to false-positive results produced compared to PCR and were discontinued and used only for sero-epidemiology studies (18). Pressure on the government to control the spread of COVID-19 led to an appreciation of the role of the laboratory in testing for diagnosis (17). Government and partners channeled funds toward the procurement of laboratory consumables and PCR equipment. Unfortunately, testing capacity was limited as many laboratories needed training and provision of PCR equipment. The relief from leveraging on a few GeneXpert machines was short-lived since they have few testing channels and could only test a few samples per day. GeneXpert cartridges were not available for nearly 2 months, presenting a drawback to efforts made to decentralize PCR leveraging on GeneXpert machines, in the provincial and mission hospitals. Furthermore, the efforts were overtaken by events as demand for testing increased, and at the same time, the global supply pipeline was overwhelmed and could not meet the demand or timely supply of ordered goods (19). Pressure mounted and increased the turnaround times to more than 48 h, and there was an urgent need for immediate intervention. The introduction of the Ag-RDT testing platform was timely in enhancing access and accelerated testing services to serve both institutional and community testing needs.

The main objective of implementing this program was to decentralize testing, increase accessibility to COVID-19 testing for a large population, mainly at the primary level and reduce turnaround time (20, 21). At the beginning of the pandemic, only one national laboratory could test for COVID-19 as demanded by institutions. The adoption of the Ag-RDTs by the lab pillar headed by the Director of laboratory services (MoHCC) was a game-changer. Ag-RDT was appropriate for our setting as it enabled the system to effectively utilize available human resources, as non-laboratory professionals could use the Ag RDTs with appropriate training (15, 22). In our setting, we experienced an increase in the number of tests carried out, mainly due to the contribution of the Ag-RDT, as this did not affect the number of tests performed by PCR. Decentralization improved access to service delivery, provided testing at local health facilities, and improved testing coverage (21). Improved turnaround times brought about early detection of COVID-19 facilitating implementation of containment measures timeously (20).

The formation of the four tiers was key in COVID-19 surveillance as this was a step-by-step intervention and decision making as the pandemic progressed through the four scenarios of transmission from containment phase 1 to community widespread transmission in phase 4. This is evidence of the many meetings, decisions, and public health interventions considered, along with the story of the evolving pandemic. Extensive testing and integration of SARS-CoV-2 infection into the routine testing system are paramount for disease surveillance and monitoring. End-to-end interim guidance has been produced by WHO, which states the integration of SARS-CoV-2 and influenza virologic and genomic surveillance from sentinel site case enrolment (23). Continual disease surveillance through genomic sequencing will allow timeous monitoring of disease trends, including being alert on any possible introduction of new SARs- CoV-2 variants that can threaten the population.

### Implications

- The broader implication of decentralizing is that as good as it is to increase access to testing, Ag-RDT testing may hinder the referral of PCR samples necessary for the representative genomic sequencing surveillance system that the country is establishing.
- Decentralization of testing will need a system to maintain quality testing processes.
- Decentralization reduces requirements for transportation of samples and communication of results, thereby reducing the costs related to the tests both for the patient and the system.
- Decentralized testing is an additional burden to the HCW at primary level facilities and will require recognition and compensation.

### Lessons learned for future applications

- The quick adoption of the WHO guidelines, the review of the National COVID-19 testing strategy, and the development of the implementation guideline were important in accelerating the implementation plan.
- The strong collaboration between MoHCC and partners for both financial and technical support through the lab pillar meetings, including the private sector's involvement resulted in quick decision making, resource mobilization and timely response.
- Partner involvement was key in setting up trainings and providing technical support in adopting new testing platforms and procedures across the testing laboratories and testing centers.
- Trained staff were able to cascade training to site-level through carrying out in-house trainings to other staff.
- The implementation of a comprehensive Quality Assurance programme including method selection, training, competence assessment, internal quality control (IQC), and External Quality Assessment (EQA) was instrumental in assuring quality reliable results.
- Reducing the trainings to a single day at the site level meant covering more sites within a short space of time.
- The unavailability of a viable data management solution for Ag-RDT did not hinder implementation. Therefore, the use of a paper-based and WhatsApp daily reporting platforms enabled easy data sharing from remote sites to the national level.
- Demand creation through stakeholder sensitization meetings was key in accepting and implementing antigen testing.
- Expedited decentralization, task shifting within financial / employment constrained resources setting were crucial in increasing access to testing.
- Filling the Knowledge gap was instrumental in reducing the fear of infection during testing, changing negative attitudes, and enhancing good practice.
- For timely intervention at all levels, feedback through weekly reporting on training progress and monitoring in the Provincial Health Executive (PHE) meetings were carried out consistently.

### Acknowledgment of any conceptual or methodological constraints

Conceptual constraints observed in this intervention included shortened training time and insufficient practical exposure since training was reduced to 1 day. However, it should be noted that the country leveraged the nurses trained in HIV rapid testing and lab personnel who were pioneers in testing using COVID-19 Ag testing. Furthermore, the province selected participants for training, and some of the trainees may not have been directly involved in patient testing. There was also high turnover among trained cadres either due to rotation across departments, attrition, or trained personnel going on leave and thus affecting COVID-19 testing.

## Conclusion

The decentralizing and expanding testing approach was successfully implemented and achieved its objectives. The Ag-RDT testing was instrumental in increasing overall testing capacity, strengthening rapid response activities such as disease surveillance and control, case management, and timely national data reporting for decision making. Emergency response interventions need continuous refresher training, mentorship, and monitoring to maintain standardized quality testing processes. Future workaround use of Ag RDTs in Zimbabwe should help track the evolution of variants by setting up a system that enforces referral of selected samples for genomic surveillance.

## Data availability statement

The raw data supporting the conclusions of this article will be made available by the authors, without undue reservation.

## Ethics statement

Ethics approval and written informed consent were not required for this study in accordance with national guidelines and local legislation.

## Author contributions

MG-M, KS, AC, SZ, LC, RS, and AG: contributed to study conception, design, and implementation of the study. MG-M, AC, KS, SZ, LS, LC, RS, and AG: data collection, data analysis, manuscript writing, and revisions. All authors contributed to the article and approved the submitted version.

## Funding

This funding was provided by the following partners, CHAI and WHO through the African Development Bank (AFDB) fund.

## Conflict of interest

The authors declare that the research was conducted in the absence of any commercial or financial relationships that could be construed as a potential conflict of interest.

## Publisher's note

All claims expressed in this article are solely those of the authors and do not necessarily represent those of their affiliated organizations, or those of the publisher, the editors and the reviewers. Any product that may be evaluated in this article, or claim that may be made by its manufacturer, is not guaranteed or endorsed by the publisher.

## Approval

Through the laboratory directorate, the Ministry of Health and Child Care approved the development of this manuscript.
